# The effect and acceptability of tympanometry and pneumatic otoscopy in general practitioner diagnosis and management of childhood ear disease

**DOI:** 10.1186/s12875-014-0181-x

**Published:** 2014-12-12

**Authors:** Penelope Abbott, Sara Rosenkranz, Wendy Hu, Hasantha Gunasekera, Jennifer Reath

**Affiliations:** Department of General Practice, University of Western Sydney, Locked Bag 1797, Penrith, NSW 2751 Australia; Department of Human Nutrition, Kansas State University, Manhattan, USA; Medical Education Unit, School of Medicine, University of Western Sydney, Sydney, Australia; Children’s Hospital at Westmead Clinical School, University of Sydney, Sydney, Australia

**Keywords:** Otitis media, Otoscopy, Tympanometry, General practitioners, Diagnosis, Ear diseases

## Abstract

**Background:**

Tympanometry and pneumatic otoscopy are recommended for diagnosis of otitis media, but are not frequently used by general practitioners (GPs). We examined how, after targeted short training, GP diagnosis and management of childhood ear disease was changed by the addition of these techniques to non-pneumatic otoscopy. We further explored factors influencing the uptake of these techniques.

**Methods:**

Between 2011 and 2012, we used a crossover experimental design to determine associations between tympanometry and pneumatic otoscopy and the GP diagnosis and management of ear disease in children aged 6 months to 6 years. GPs recorded a diagnosis and management plan after examining ears using non-pneumatic otoscopy, and another after using either tympanometry or pneumatic otoscopy. We compared diagnosis, prescription of oral antibiotics and planned GP follow-up at these two steps between the tympanometry and pneumatic otoscopy groups. We interviewed participants about their views regarding these techniques and analysed these data thematically.

**Results:**

Thirteen GPs recorded 694 ear examinations on 347 children: 347 examinations with non-pneumatic otoscopy; then 196 using tympanometry; and 151 using pneumatic otoscopy. Tympanometry was more likely to be associated with changes in diagnosis (χ ^2^ = 28.64, df 1, p < 0.001) and planned GP follow-up (χ ^2^ = 9.24, df 1, p < 0.01) than pneumatic otoscopy. Change in oral antibiotic prescription was no different between the two techniques. GPs preferred tympanometry to pneumatic otoscopy, but cost was a barrier to ongoing use. Pneumatic otoscopy was considered the more difficult skill. GPs were not convinced that the increased detection of middle ear effusion afforded by tympanometry and pneumatic otoscopy resulted in benefit to general practice patients.

**Conclusion:**

Tympanometry was more likely than pneumatic otoscopy to change GP diagnoses and follow-up plans of childhood ear disease. Tympanometry may require less training than pneumatic otoscopy. GPs preferred tympanometry due to ease of use and interpretation; however, perceived high cost inhibited their intent to use it in the future. Training, cost and perceived lack of patient benefit are barriers to the use of tympanometry and pneumatic otoscopy in general practice.

## Background

Otitis media (OM) is a common reason for general practitioner (GP) consultations and for antibiotic prescriptions [1-3] and creates a large health and cost burden to communities [4,5]. It can lead to significant complications and affect children’s education and quality of life [2]. The most common forms of OM are acute otitis media (AOM) and otitis media with effusion (OME), both of which are characterised by the presence of middle ear effusion [2]. The presence of symptoms and signs of acute infection in AOM is the key differential between these forms of OM [6,7].

In Australia, otitis media is usually diagnosed and managed by GPs. A GP referral is required for appointments with paediatricians or otolaryngologists under the universal health insurance scheme. Audiologists are accessed privately or after GP referral.

Current Australian guidelines recommend that children with AOM should be treated with antibiotics on first presentation if they are aged less than 6 months, have marked vomiting or fever, or are under 2 years and have bilateral disease [8]. Early antibiotic treatment is recommended for all Aboriginal and Torres Strait Islander children living in rural and remote communities where chronic suppurative otitis media is prevalent [9]. Antibiotics are generally not indicated for OME [8,9]. Recommended follow up of uncomplicated OME is to repeat the examination in 3 months, and if OME is still present, referral for audiometry is needed, potentially followed by otolaryngologist review. Aboriginal children with uncomplicated AOM should be reviewed in 1 week [9], and other children are generally followed up according to their symptoms only, with little evidence that follow up after uncomplicated AOM is useful [7].

GP management of OM often falls outside best practice guidelines [2,10-12]. Challenges in accurate diagnosis of OM may contribute to this. There is evidence GPs tend to over-diagnose AOM and under-diagnose OME [13,14]. As best practice management differs between these two conditions, over-diagnosis of AOM may lead to unnecessary antibiotic prescription with the resultant risk of side effects and community antibiotic resistance [7,12,15]. Failure to diagnose OME may lead to suboptimal management such as inadequate hearing assessment and follow up [16].

Tympanometry and pneumatic otoscopy are recommended for diagnosis of otitis media, but are not frequently used by general practitioners [7,13,17-19]. Recent clinical practice guidelines have stated that primary care clinicians should not diagnose AOM in children who do not have a middle ear effusion as demonstrated by tympanometry or pneumatic otoscopy because non-pneumatic otoscopy is inaccurate [7].

The reasons why GPs have not embraced the use of tympanometry or pneumatic otoscopy have not been well elucidated. It has been suggested that GPs do not consider confirming the presence of effusion to be an important factor when diagnosing AOM [20] and that GPs lack training in use of these techniques [21-24]. Training in tympanometry or pneumatic otoscopy is not standard within Australian general practice.

There is little research evidence around what type and extent of training may be required to ensure GP skill and confidence in performing and interpreting tympanometry and pneumatic otoscopy [18,22,25]. A 6 hour course in tympanometry and OM was reported to be effective in increasing the knowledge and skills of GPs and practice nurses in Denmark, and participating GPs reported the use of tympanometry changed their management of middle ear disease when they were surveyed 6 weeks after the course [26]. There has been little other research into whether the use of tympanometry and pneumatic otoscopy is practical and acceptable to GPs, and how they impact on diagnosis and management of OM in general practice.

We have previously reported the implementation and effectiveness of a workshop to train GPs in the use of tympanometry and pneumatic otoscopy [22]. The aim of the current study was to examine whether or not GP diagnosis, antibiotic therapy and follow-up of OM in childhood were changed by the use of tympanometry and pneumatic otoscopy, including comparison of these two diagnostic techniques, and to investigate influences on GP uptake of the techniques.

## Methods

The study took place between 2011 and 2012. Approval was provided by the University of Western Sydney human research ethics committee (H8960) and the ethics committee of the Aboriginal Health and Medical Research Council of New South Wales (768/10).

### Training in pneumatic otoscopy and tympanometry

We invited GPs in western Sydney to attend a 3-hour workshop aiming to improve their skills in the use of tympanometry and pneumatic otoscopy. The workshop was advertised through email invitations and in a regular regional general practice newsletter. The workshop comprised a large group presentation on the diagnosis and management of OM in children, followed by small group skills-based training in tympanometry and pneumatic otoscopy, facilitated by an ENT surgeon and an audiologist. Participants were given links to resources including an online training resource [25]. We have previously described the methods and training outcomes [22].

### Participants

We invited the 23 GPs who completed the workshop to participate in this study. We provided follow-up training and support as necessary, including 1-2 practice visits to all participants by a GP (PA) skilled in the use of these techniques. Participants could elect to have a 2-week lead-in time to familiarise themselves with the techniques prior to commencing the study. Of the 23 GPs who attended the workshop, 13 GPs (males = 7, females = 6) elected to participate in the study. GPs completed a demographic questionnaire prior to commencement. The GPs estimated the number of children aged under 6 years that they saw in an average week to range from 3 to 60 children, with a median of 18. Characteristics of GP participants are shown in Table 1.

**Table 1:** **Characteristics of GPs participants**

**Characteristics**	**N (%)**
Gender	
Male	7 (54)
Female	6 (46)
Age	
30-39	3 (23)
40-49	3 (23)
50-59	6 (46)
60+	1 (8)
Undergraduate training	
Australian graduate	8 (62)
International medical graduate	4 (31)
Not stated	1 (8)
Years since graduation	
6-15	4 (31)
16-25	3 (23)
26+	6 (56)
Clinical sessions worked /week	
2-4	2 (31)
5-7	4 (31)
8-10	7 (54)
Works in a GP training practice	
Yes	10 (77)
No	2 (15)
Not stated	1 (8)
No. GPs in main practice	
2-3	4 (31)
5-9	3 (23)
10-16	6 (46)
No. children seen in average week *(range 3-60, median 18)*	
Less than 10	4 (31)
10 - 29	4 (31)
30+	4 (31)
Not stated	1 (8)

### Equipment

GPs were provided with the same model of portable tympanometer (Amplivox Otowave Hand Held Portable Tympanometer 102), though one GP also used their own tympanometer for some examinations. GPs used their own otoscopes, and we provided the appropriate insufflator bulb to enable pneumatic otoscopy.

### Study design

We used a crossover experimental study design to investigate the effect of the use of tympanometry or pneumatic otoscopy in addition to non-pneumatic otoscopy on the diagnosis and management of OM. We examined management in terms of the therapy given, in particular use of oral antibiotics, and any scheduled follow up of the child, either by the GP or through referral to an audiologist or otolaryngologist. We asked GPs to provide study data every time they examined the ears of a child aged 6 months to 6 years if they believed an ear examination was indicated as part of their standard practice. GP participants were asked to note the purpose of the examination, namely for ‘diagnosis’, ‘screening’ or for ‘follow-up’ of known ear disease.

**Figure 1:**
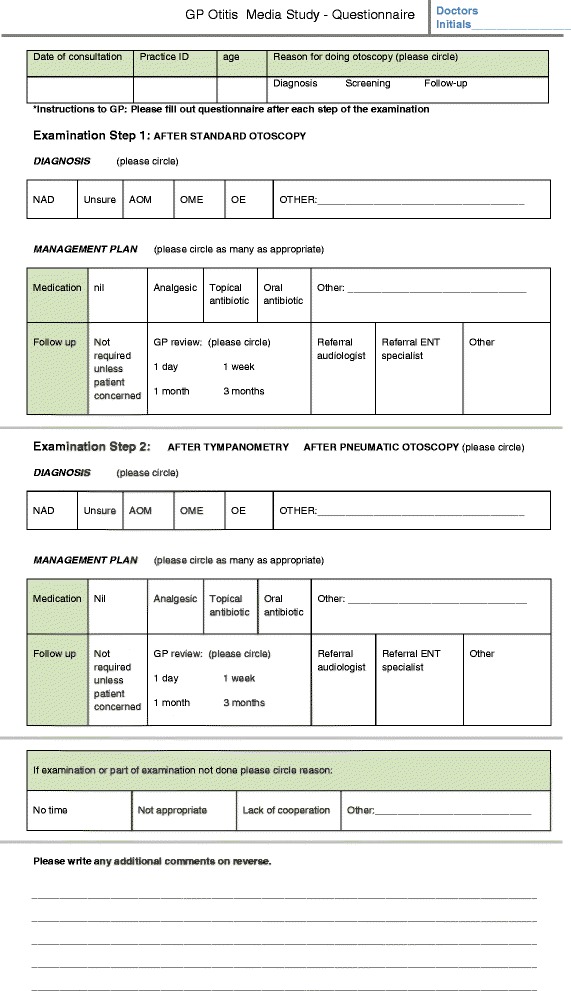
**Data collection questionnaire.**

GPs all performed non-pneumatic otoscopy (‘Step 1’) and documented their initial diagnosis, therapy and follow-up plan on a one-page datasheet (Figure 1) based on this assessment alone. Then they repeated the ear examination using either tympanometry or pneumatic otoscopy (‘Step 2’), again noting their diagnosis, therapy and follow-up plans. We did not require GPs to note more than one diagnosis in the child. Thus, if there were different otoscopic findings in each ear, GPs were asked to select the diagnosis that was the most significant within that consultation in guiding subsequent therapy and follow up. We did not collect any identifying data on the children being examined.

In Step 2, GPs performed either tympanometry or pneumatic otoscopy according to their assigned technique at the time of the examination. Participating GPs were assigned to these two arms based on the availability of the equipment when the GP was ready to start the study, and on the need to allocate equal numbers of GPs to each arm of the study. We asked the GPs to cross over to the alternate diagnostic technique arm after a period of 1 month of data collection or 25 ear examinations and then continue with their data collection.

### Outcome measures

The outcome measures related to the diagnosis, therapy and planned follow up which GPs documented at both study steps. Diagnosis was categorised as one of the following: no abnormality; unsure; acute otitis media; and otitis media with effusion. Therapy referred to prescription of oral antibiotics or recommendation for analgesic therapy. Planned follow up comprised recommendation for GP review or referral to an audiologist or an otolaryngologist.

### Statistical analyses

Statistical analysis was undertaken using SPSS (version 18.0; SPSS Inc., Chicago, Ill.). We used chi-square analyses or Fisher’s exact test to determine associations between use of tympanometry or pneumatic otoscopy and change in diagnosis, prescription of antibiotic therapy, and GP follow up between Step 1 and Step 2. We examined factors associated with increased likelihood for change in diagnosis between Step 1 and Step 2 using binary logistic regression. Significance was set at p < 0.05.

### Qualitative study of the usefulness of tympanometry and pneumatic otoscopy

We interviewed all participating GPs regarding their experiences and views of tympanometry and pneumatic otoscopy, including: the usefulness of the techniques; their training needs; and whether current guidelines on OM diagnosis were applicable in a GP setting. The interviews took place within 1 month of the GP concluding the quantitative arm of the study. The interviews were audiotaped and transcribed verbatim, and two members of the research team (PA and WH) undertook the qualitative data analysis using a framework approach [27]. The thematic framework was developed from predetermined questions related to the aims of the study as well as themes which arose from the participants themselves. The two researchers coded separately into the existing and emergent categories, and final themes were resolved through iterative review.

## Results

GPs performed a total of 694 ear examinations on 347 children. All 347 children were initially examined with non-pneumatic otoscopy (Step 1). Then, 196 were examined with tympanometry; and 151 with pneumatic otoscopy (Step 2). Four GPs reported examinations on between 1 and 7 children each, and nine reported examinations on between 19 and 50 children. Two GPs declined to collect data using pneumatic otoscopy and therefore only contributed data for Step 1 and for tympanometry as Step 2. They had both received the workshop training and follow-up practice visit but, after the two-week lead-in period which was designed to increase confidence with the technique, they were not willing to proceed to data collection for pneumatic otoscopy. GPs performed otoscopy for diagnostic purposes in 60% of children, for screening in 17%, and for follow up in 14%, with no reason documented in the remaining 9%.

### Comparison of GP diagnoses and management plans after addition of tympanometry and pneumatic otoscopy

Table 2 shows the frequency of diagnoses, therapy (including oral antibiotic therapy and symptomatic treatment) and follow-up plans after non-pneumatic otoscopy, and how these differed after tympanometry and pneumatic otoscopy. When comparing management between the two steps of the study, we only considered change in oral antibiotic therapy and decisions that a GP review was needed, regardless of the planned timing of the review.

**Table 2:** **Diagnosis and management plans including changes after tympanometry or pneumatic otoscopy**

		**Children examined n (%)**				
		**After non-pneumatic otoscopy –all examinations at step 1**	**Examinations grouped according to the diagnostic technique used at step 2**			
			**Tympanometry**		**Pneumatic otoscopy**	
		**n = 347 (100%)**	**n = 196 (56%)**		**n = 151 (44%)**	
			***Step 1***	***Step 2***	***Step 1***	***Step 2***
			***(non-pneumatic otoscopy only)***		***(non-pneumatic otoscopy only)***	
Diagnosis	No abnormality	153 (44)	78 (40)	72 (37)	75 (50)	78 (52)
	Unsure	50 (14)	30 (15)	18 (9)	20 (13)	15 (10)
	Acute otitis media	56 (16)	31 (16)	17 (9)	25 (17))	25 (17)
	Otitis media with effusion	63 (18)	44 (22)	60 (31)	19 (13)	20 (13)
	Other	20 (6)	9 (5)	27 (14)	11 (7)	8 (5)
	Missing data	5 (1)	4 (2)	2 (1)	1 (<1)	5 (3)
Therapy * more than one response possible	Not required	199 (57)	110 (56)	108 (55)	90 (60)	88 (58)
	Oral antibiotic	66 (19)	35 (18)	34 (17)	31 (21)	26 (17)
	Analgesic	36 (10)	18 (9)	16 (8)	18 (12)	17 (11)
	Missing data	42 (12)	30 (15)	32 (16)	12 (8)	15 (10)
Planned follow up *more than one response possible	Not required	165 (48)	85 (43)	76 (39)	80 (53)	80 (53)
	GP review needed	124 (36)	76 (39)	91 (46)	48 (32)	46 (31)
	Referral audiologist	4 (1)	1 (<1)	2 (1)	2 (1)	5 (3)
	Referral otolaryngologist	4 (1)	3 (2)	7 (4)	1 (<1)	2 (1)
	Missing data	51 (14)	31 (16)	26 (13)	20 (13)	18 (12)

There was no significant difference at Step 1 in terms of GP diagnosis (χ^2^ = 10.6 *df* = 5, *p* = 0.06), therapy with an oral antibiotic (χ^2^ = 0.07, *df* = 1, p = 0.80) and planned follow-up by GP (χ^2^ = 2.66, *df* = 1, *p* = 0.10) between children who went on to have tympanometry versus pneumatic otoscopy.

1. Change in diagnosis

Tympanometry was more likely than pneumatic otoscopy to be associated with a change in diagnosis between Steps 1 and 2 (χ^2^ = 28.64, df = 1, p < 0.01) (Table 3). At step 1 a diagnosis of ‘no abnormality detected’ (‘NAD’) was more likely to be changed to a diagnosis of ‘OME’ after the use of tympanometry (p < 0.01).

**Table 3:** **Changes in diagnosis, oral antibiotic therapy and GP review plans between Step 1 (non-pneumatic otoscopy) and Step 2 by technique used**

**Change between step 1 and 2**	**Tympanometry**		**Pneumatic otoscopy**			**Significance**
						**χ** ^**2**^ **and p value**
	**Yes**	**No**	**Yes**	**No**	**Incomplete data**	
Any change in GP diagnosis	96	93	32	113	33	28.64, df 1, p < 0.01
*Change from diagnosis of NAD at Step 1 to OME at Step 2*	*10*	*44*	*0*	*67*		*p < 0.01* ^*†*^
*Change from AOM at Step 1 to NAD at Step 2*	*5*	*26*	*1*	*24*		*p = 0.21* ^*†*^
*Change from OME at Step 1 to NAD at Step 2*	*10*	*28*	*4*	*14*		*0.11, df 1, p = 1*
*Change from Unsure at Step 1 to NAD, AOM or OME at Step 2*	*24*	*6*	*13*	*7*		*1.4, df = 1, p = 0.33*
*Change from NAD, AOM or OME at Step 1 to Unsure at Step 2*	*15*	*139*	*9*	*110*		*0.40, df 1, p = 0.53*
Change in decision to prescribe oral antibiotic	12	136	5	122	72	2.05, df 1, p = 0.15
Any change in planned GP review	33	122	10	114	87	9.24, df 1, p < 0.01
*Change from no plan for GP review at Step 1 to GP review plan at Step 2*	*21*	*63*	*5*	*72*		*10.16, df 1, p < 0.01*
*Change from GP review plan at Step 1 to no plan for GP review at Step 2*	*13*	*57*	*7*	*39*		*0.22, df 1,p = 0.64*

*†Fisher’s exact test.*

GP = General practitioner (primary healthcare practitioner).

AOM = Acute otitis media, OME = Otitis media with effusion, NAD = No abnormality detected.

Both techniques appeared to assist some GPs to make a diagnosis when they were initially unsure, with 24/30 in the tympanometry moving from a diagnosis of ‘unsure’ to either ‘NAD’, ‘AOM’ or ‘OME’ and 13/20 in the pneumatic otoscopy arm; the difference was not significant between the two techniques (p = 0.33). However, several changes in diagnoses in both arms were consistent with persistent diagnostic uncertainty. For example, the change from a diagnosis of NAD, AOM or OME to ‘unsure’ occurred similarly in both the tympanometry (15/154) and pneumatic otoscopy (9/119) arms (χ^2^ = 0.40, df = 1, p = 0.53).

The unadjusted odds ratios for technique (tympanometry versus pneumatic otoscopy), initial diagnosis, patient age and reason for otoscopy are shown in Table 4. Using the adjusted model, the use of tympanometry increased the odds of changing diagnosis from Step 1 to Step 2 by three times compared to pneumatic otoscopy (AOR = 3.33, 95% CI 2.04-5.55). Patient age, reason for otoscopy, and step 1 diagnosis, were not associated with change in diagnosis.

**Table 4:** **Factors predicting a change in diagnosis at Step 2 (tympanometry or pneumatic otoscopy used)**

	**Unadjusted**		**Adjusted**		
**Variable**	**OR**	**95% CI**	**OR**	**95% CI**	**Nagelkerke R2**
Technique (tympanometry or pneumatic otoscopy)	3.70*	2.22-5.88	3.33*	2.04-5.55	0.12
Patient age (months)	0.99	0.98-1.00	0.99	0.98-1.01	
Reason for otoscopy	0.88	0.65-1.20	0.87	0.63-1.20	
Step 1 Diagnosis	0.96	0.82-1.12	0.99	0.83-1.18	
Nagelkereke R2 = 0.11 (full model)					

* indicates significance at the p < 0.05 level.

OR = odds ratio, 95% CI = 95% confidence interval.

2. Change in management

The change in prescribing oral antibiotics between steps 1 and 2 was no different for those who were examined using tympanometry versus pneumatic otoscopy at step 2 (χ ^2^ = 2.05, *df* = 1, *p* = 0.15) (Table 3).

The use of tympanometry increased the likelihood that GPs would plan to follow-up the child, compared to pneumatic otoscopy (χ ^2^ = 9.24, *df* = 1, p < 0.01) (Table 3). There was a strong correlation between a change of diagnosis and a change in planning GP review between steps 1 and 2 (χ ^2^ = 63.23, *df* = 1, *p* < 0.01).

Changes in planned referral to an audiologist or otolaryngologist could not be reliably assessed due to small numbers.

### GP views on tympanometry and pneumatic otoscopy

All 13 GPs participated in interviews, either face to face (n = 12) or by telephone (n = 1), and interview duration ranged from 10 – 35 minutes. Key findings are illustrated in Table 5. Most GPs found both tympanometry and pneumatic otoscopy were acceptable to carers and children, although some GPs stated they preferred not to use pneumatic otoscopy as children sometimes found it uncomfortable. Some GPs thought tympanometry was particularly useful for communicating with carers about ear disease, offering tangible ‘proof’ to parents of the GP diagnosis and support of the management plan.

**Table 5:** **Participant views on the use of tympanometry and pneumatic otoscopy**

Acceptability to patients	Both of them were easy to do. I think most of the patients like especially the tympanogram … it was very easy to show a parent, “Here’s the little graph, it’s flat, it needs to have a peak, its peak is supposed to be in the box. Come back in a couple of weeks, we’ll do it again and we’ll make sure that the peak’s in the box”. And parents responded really well to that, it was something they could see, it was a change that could be measured. (GP 9)
	The kids tend to not quite like that [pneumatic otoscopy] as well, it caused more discomfort so why do I want to keep causing discomfort to every kid. (GP 5)
Ease of use of tympanometry and pneumatic otoscopy	I found the pneumatoscope harder than the tympanogram … Sometimes I could see the drum moving beautifully, sometimes it just wouldn’t move, and yet I didn’t know whether that was technique or pathology…Whereas, the tympanogram tells you if you’ve achieved a seal, and so you’re more confident in your technique. (GP 12)
	The technical side of using the tympanometer is not that tricky … but it is also about interpreting the results. Sometimes it is not quite clear cut. (GP 5)
	People that have got very little training at all could use it [tympanometry] quite professionally because it’s really easy. It basically does everything for you, and you know, you know which one to press for the next step and so and so. (GP 10)
GPs attitudes to whether detecting effusions is essential in diagnosis of AOM	Not acute otitis media, I guess a lot of it’s clinical, in the story, and then the drum looking red. (GP 12, discussing whether pneumatic otoscopy or tympanometry are needed for diagnosis of AOM)
	[Tympanometry or pneumatic otoscopy] has to be part of the examination. Just looking at the eardrum is not enough to diagnose it unless they come with a terribly red eardrum … but I myself don’t think that this enough. It is just a guess. (GP 7)
	I didn’t find them helpful [for diagnosis of AOM)….I mean if it’s really acute, you know, it’s like staring me in the face. (GP 2)
Diagnosis of OME increased	If I hadn’t done the tympanogram I might have just said, “See you later”, you know? It’s hard to appreciate middle ear pathology, I think, just from looking. (GP 12)
	I thought it (tympanometry) changed my management. Follow ups. Otherwise I would have told them that it looks alright. Because if it is flat, I ask them to come back and then get it checked again so, it is a lot of more follow up. (GP 7)
	There’s no good cone of light, and there’s this retraction, but the Tymp was perfect. Which means it’s better not to depend on your eyes.(GP 4)
Barriers to increasing use of tympanometry and pneumatic otoscopy in general practice	[The tympanometer] is too expensive, so I don’t, I just don’t think I will buy that one, the cost is the issue for that one - and not much rebate. (GP 3)
	When we invest extra time to actually do additional tests you want to gain something beneficial for the patient management. It [pneumatic otoscopy] didn’t feel like it is worth the additional, time investment to do that because even when there is an outcome most of the time I am not quite sure whether it is reliable or not. (GP 5)
	It [tympanometry] actually didn’t change my management … If ENT specialists use that it may change their management but with me, no I didn’t change my management … Your clinical judgment is the most important in my view, so what you can see through your eye with opthalmoscope is the one which is more, is sufficient and the presentation, duration of the symptoms, history and examination. (GP 13)
	I am not too sure that all the ear drums need to be referred … How accessible is it to send them to hearing test, how often do you send them for a hearing test? When you send to the ENT, look at the cost issue and the waiting time issue and the impact on the family. (GP 7)
	I suppose there needs to be evidence that widespread use would improve outcomes if all they [pneumatic otoscopy and tympanometry] do is pick up stuff that the natural course of it is resolution. (GP 2)

There was a preference for tympanometry based on ease of use and interpretation by all participants. GPs believed more training and experience was needed to become confident with pneumatic otoscopy than tympanometry, and some GPs ‘gave up’ on pneumatic otoscopy. The training provided in this study was reported by most GPs as adequate to allow them to incorporate tympanometry in their practice, the machine being largely self-explanatory and easy to use. GPs could teach themselves how to perform tympanometry after the relatively brief introduction to the equipment, with the main challenge being interpretation of the tympanograms, which required them to refer back to written information they were given during training. Pneumatic otoscopy was seen as the more difficult technical skill. The most important barrier to using pneumatic otoscopy perceived by GPs in this study was their uncertainty as to whether there was true drum immobility or whether their technique was simply inadequate.

Most GPs stated that both tympanometry and pneumatic otoscopy could assist in diagnosis of OM without creating an undue time burden within the consultation, however, this did not translate into a strong intention to use the techniques in the future. While some GPs believed the techniques had improved the accuracy of their diagnoses of OM, for others uncertainty was created when the findings did not agree with their clinical judgement, such as evidence of an effusion in a normal looking ear. The techniques were seen to have most value in the diagnosis and monitoring of OME. There was less agreement that the techniques were useful in diagnosis of AOM. Several GPs stated that neither tympanometry nor pneumatic otoscopy were needed for the diagnosis of AOM, as they were guided by other symptoms and signs of acute inflammation such as pain and a red eardrum.

Some GPs expressed ambivalence as to whether current guidelines recommending the use of tympanometry and pneumatic otoscopy were practical in the GP setting. They were concerned they may be detecting effusions which were not clinically important as general practice differed from an otolaryngology or paediatric setting, and follow up may create unnecessary health costs to patients and the community through increased GP follow up and referral to audiologists and otolaryngologists. Many were also unclear on the significance of negative pressure (Type C) tympanometry readings in general practice, reported to have been a common finding in ear examinations which could potentially generate unnecessary GP follow ups. Type C tympanometry readings may have been particularly implicated in the diagnoses of ‘other’ according to GP report. We did not capture the tympanometry or pneumatic otoscopy findings and so cannot report on the frequency of these findings.

Most GPs (9 of 13) said they would choose to continue to use tympanometry, however they perceived the cost of the tympanometer to be prohibitive given the low reimbursement for this service in the Australian universal healthcare system. One participant expressed a strong intention to use tympanometry in the future and another to use both tympanometry and pneumatic otoscopy. One of those GPs intended to purchase a tympanometer for their practice and the other had access to a tympanometer which she had not previously used.

## Discussion

We have shown that tympanometry and pneumatic otoscopy changed GP diagnosis and planned follow up of children with ear disease compared to diagnoses and plans based on non-pneumatic otoscopy alone. However, tympanometry had a greater effect than pneumatic otoscopy and may have been more effective at detecting middle ear effusions in this study.

Tympanometry was more likely than pneumatic otoscopy to prompt a decision to follow up a child who would otherwise not have had planned review by the GP. In a previous study with GPs in Denmark, tympanometry changed the GP diagnosis of OM made on the basis of non-pneumatic otoscopy in about one quarter of children, though not to the level of statistical significance, but did not change planned follow up [28]. Our interview data suggested that some of the increase in planned GP follow up in the tympanometry arm was related to increased detection of OME. However, it was also evident some of the increased follow up may reflect the reportedly high number of negative pressure (Type C) tympanometry readings and uncertainty by some GPs as to the management of this finding.

Most GPs reported tympanometry to be easy to master and useful when assessing children’s ears after the short training intervention, whereas pneumatic otoscopy was seen as the more difficult skill and was less likely to be regarded positively by GPs than tympanometry. Notably two GPs declined to proceed to using pneumatic otoscopy after the training provided, whereas GPs quickly gained confidence in their tympanometry skills despite relatively brief training. However, most reported they were unlikely to use tympanometry, citing cost as a barrier. The small benefit payable for tympanometry in the Australian universal health care scheme at the time of the study meant that approximately 250 tympanometry examinations would be needed to recoup the cost of one tympanometer.

Most participants agreed that both tympanometry and pneumatic otoscopy could assist in diagnosing OM and that current guidelines recommending their use in primary care were reasonable. However, it seems GPs’ appreciation of the value of the techniques was theoretical only, as 11 out of 13 GPs intended to continue to use only non-pneumatic otoscopy in ear examinations at the end of the study. The evaluation of the training workshop which preceded the current study showed similar findings in that workshop participants had increased self confidence in the use of tympanometry and pneumatic otoscopy but no increase in their intention to use either technique in the future [22].

A barrier to the use of both techniques was a belief that the techniques did not provide essential information for their diagnosis and management of otitis media. This finding aligns with a Finnish study showing that GPs are likely to base their diagnosis of AOM on symptoms and the colour of the tympanic membrane more than the movement and position of the ear drum indicating presence of a middle ear effusion [20]. In addition, it appears that GPs are yet to be convinced that the increased detection of OME afforded by tympanometry and pneumatic otoscopy equates to patient benefit in the general practice setting, despite this being a core recommendation of current guidelines [7]. This lack of belief in patient benefit from tympanometry and pneumatic otoscopy is another potential explanation for the current underutilization of tympanometry and pneumatic otoscopy by GPs in Australia and internationally.

Both tympanometry and pneumatic otoscopy are known to improve accuracy in diagnosis of OM. Our study provides evidence that tympanometry may be more readily introduced into general practice. However, improving GP diagnosis of OM will not automatically improve the management of OM and patient outcomes. Most cases of OM in Australian general practice still appear to be treated with antibiotics despite best practice recommendations against this. Antibiotics are reported to have been prescribed in 82% of diagnoses of AOM and 65% of diagnoses of ‘chronic otitis media’ between 2000-2007 in Australia, with little evidence of more recent decrease in antibiotic prescription rates [5,29]. Conceivably, an increase in GP use of tympanometry and pneumatic otoscopy could *increase* the number of OM diagnoses and lead to more antibiotics being prescribed. Similarly, more diagnoses of OM after routine ear examinations in low risk children could prompt unnecessary GP follow up and otolaryngologist referral without clear guidance. For example, current guidelines do not recommend routine follow up after uncomplicated AOM in children who are not Aboriginal and Torres Strait Islander [7,9]. The acceptability of this recommendation to GPs is unclear, particularly as GPs are also advised not to give antibiotic treatment to this group, and yet usually do so. In addition, current guidelines do not clearly address management and follow up of some scenarios which our research suggests could be more commonly encountered with wider GP use of tympanometry in particular, namely, OM detected through opportunistic ear screening of asymptomatic low risk children and negative pressure tympanograms.

Training in the techniques of tympanometry or pneumatic otoscopy is needed for medical students, general practitioners and practice nurses. However increased detection of OM by promoting tympanometry and pneumatic otoscopy in general practice must be accompanied by training and clinical guidelines which address the common clinical issues faced by GPs, so as to ensure good patient outcomes and to avoid increased health costs and patient burden.

Our study has several strengths including that it was based in a ‘real life’ GP setting, compared the techniques after a similar duration of training, and used a cross over design to compare the effect of each technique. Our study also has some limitations. Our findings may reflect weaknesses in the training and support provided to participants rather than difficulty in mastering the techniques. Given the small numbers in our sample, participants are unlikely to have been representative of the wider GP population. They may have had higher learning needs than their peers, though selection bias would be more likely to have resulted in a more motivated group who were interested in improving their management of OM in children.

## Conclusion

Tympanometry was more likely than pneumatic otoscopy to change GP diagnoses and plans to review children with otitis media. After minimal training, GPs preferred tympanometry due to ease of use and interpretation; however, the perceived high cost inhibited their intent to use it in the future. Pneumatic otoscopy was seen as the more difficult skill and GPs were less likely to plan to use it in the future. GPs may need to be convinced of the benefits of using both pneumatic otoscopy and tympanometry to more reliably detect OM in the general practice setting, and further convincing of the benefit to their patients of reliably detecting OM. Increased training and guidelines which address common clinical issues, as well as further research regarding GP acceptance of guideline recommendations, are needed to promote evidence based antibiotic use and follow up after diagnosis of OM.
